# Diagram of an Incisor Tooth

**Published:** 1883-02

**Authors:** 


*Diagram of an Incisor Tooth.*—We are in receipt of this beautiful illustration of the minute structure of an Upper Central Incisor which was published under the direct supervision of Professor Frank Abbott, M. D., and by special request of the Dental Society of the State of New York. The object was to give to the dental profession a correct idea of the minute anatomy of a tooth. If not absolutely perfect microscopically, it approximates so closely to nature that it conveys to the mind at once an intelligent understanding of the structure and relations of the organ.

The diagram represents a longitudinal section of the tooth. It is 13½ by 9½ inches in size, and is printed in six colors. It shows, clearly, Nasmyth's membrane, the enamel, dentine, cementum, and pulp, with periosteum and socket of alveolar process, covered by the gum; the blood-vessels and nerves of the pulp; the odontoblasts surrounding the pulp; the direct connection of the non-medulated nerve-fibres with the odontoblasts, and the passing of those fibres between these bodies, into the dentinal canaliculi, and the distribution of living matter in almost every direction throughout the dentine, finally reaching the interzonal layer, (interglobular space), whence it enters the enamel, to which it is more minutely but as clearly distributed.

The price is $1.00. The S. S. White Dental Manufacturing Co. Philadelphia. One of these Diagrams has also been presented to the Dental Department of the University of Maryland.

